# Sophisticated prospective studies are needed to evaluate the relationship between chronic neck pain and autonomic function

**DOI:** 10.1590/1806-9282.20250565

**Published:** 2025-09-19

**Authors:** Fulvio Alexandre Scorza, Carla Alexandra Scorza, Josef Finsterer

**Affiliations:** 1 Universidade Federal de São Paulo, Escola Paulista de Medicina – São Paulo (SP), Brazil.; 2 Neurology and Neurophysiology Center, Department of Neurology – Vienna, Austria.

Dear Editor,

We were interested to read the review article by Afshan et al. on autonomic dysregulation in patients with chronic neck pain (CNP)^1^. A total of eight studies were included in the review, although the study design was unclear in two of them^1^. A total of 12 tests were performed to assess autonomic nervous system (ANS) function (heart rate variability, electromyography, muscle perfusion, blood pressure, heart rate fluctuations at rest and in response to a handgrip, cold pressor test, deep breathing test, skin temperature, fingertip skin temperature and evaporation, and autonomic sensitivity to psychological stressors)^1^. It was found that as pain intensity and perceived disability increase in patients with CNP, autonomic dysregulation increases and that as the duration of pain symptoms increases, the ANS becomes more provoked and increasingly dysregulated^1^. The study is noteworthy, but some points should be discussed.

The first problem is that some of the tests used to investigate ANS functions are not reliable. In particular, needle electromyography is not a means of examining the ANS. What parameters have been extracted from needle electromyography to assess autonomic functions? In addition, measurement of blood pressure without provocation is not suitable for assessing autonomic functions. Measurement of skin temperature alone is also not a reliable test to assess ANS functions, as temperature may not be determined solely by the ANS. Furthermore, it is not stated what the difference is between "heart rate variability" and "heart rate fluctuations." It is also not mentioned which 12 tests were specifically used in the eight studies (only 10 are mentioned in the methods). More appropriate tests to assess autonomic function are the quantitative sensory test, the quantitative sudomotor axon reflex test (QSART), urodynamic tests, and the thermoregulatory sweat test^2^.

The second point is that the review does not explain why two studies whose design is unknown were included in the review. According to the exclusion criteria in the methods section, these two studies should have been excluded from the analysis. The review should be repeated without these two studies.

The third point is that the publication years of the studies also cover the years of the SARS-CoV-2 pandemic^1^. How many of the included patients were SARS-CoV-2 positive and to what extent did this influence the results? SARS-CoV-2 infection and SARS-CoV-2 vaccination are known to be complicated by autonomic neuropathy^3,4^.

The fourth point is that CNP can also occur independently of autonomic dysfunction and due to numerous other causes. These include psychological overload, depression, chronic stress, inability to cope with daily stress, dissatisfaction with the professional and private situation, cervical spine trauma, sedentary lifestyle or work, osteoporosis, rheumatologic diseases, myelopathy, Hirayama disease, Chiari malformations, syringomyelia, varicosities spinalis, neuromuscular disorders, drop head syndrome, bent spine syndrome, scoliosis, and skeletal deformities of the hips or spine. Have the necessary investigations been carried out to rule out all these differential causes of CNP?

The fifth point is that comorbidities and concomitant medications were not considered as alternative causes of CNP. As both can strongly influence autonomic functions, it would have been imperative to include pre-existing conditions and current medications in the analysis.

In summary, this interesting study has limitations that affect the results and their interpretation. Addressing these limitations could strengthen the conclusions and support the message of the study. All open questions need to be clarified before readers uncritically accept the conclusions of the study. Since CNP may not be related to ANS, all different causes of CNP need to be thoroughly ruled out before attributing CNP to autonomic dysfunction.

## Data Availability

The datasets generated and/or analyzed during the current study are available from the corresponding author upon reasonable request.
